# Investigation of Ferroelectric Grain Sizes and Orientations in Pt/Ca_x_Sr_1–x_Bi_2_Ta_2_O_9_/Hf–Al–O/Si High Performance Ferroelectric-Gate Field-Effect-Transistors

**DOI:** 10.3390/ma12030399

**Published:** 2019-01-28

**Authors:** Wei Zhang, Mitsue Takahashi, Shigeki Sakai

**Affiliations:** National Institute of Advanced Industrial Science and Technology, 1-1-1 Umezono, Tsukuba, Ibaraki 305-8568, Japan; zhang.wei@aist.go.jp (W.Z.); mitsue-takahashi@aist.go.jp (M.T.)

**Keywords:** EBSD, FeFET, ferroelectric, grain, orientation

## Abstract

Electron backscatter diffraction (EBSD) was applied to investigate the grain size and orientation of polycrystalline Ca_x_Sr_1–x_Bi_2_Ta_2_O_9_ (C_x_S_1–x_BT) films in ferroelectric-gate field-effect transistors (FeFETs). The C_x_S_1–x_BT FeFETs with *x* = 0, 0.1, 0.2, 0.5, and 1 were characterized by the EBSD inverse pole figure map. The maps of *x* = 0, 0.1, and 0.2 showed more uniform and smaller grains with more inclusion of the *a*-axis component along the film normal than the maps of *x* = 0.5 and 1. Since spontaneous polarization of C_x_S_1–x_BT is expected to exist along the *a*-axis, inclusion of the film normal *a*-axis component is necessary to obtain polarization versus electric field (*P*–*E*) hysteresis curves of the C_x_S_1–x_BT when the *E* is applied across the film. Since memory windows of FeFETs originate from *P*–*E* hysteresis curves, the EBSD results were consistent with the electrical performance of the FeFETs, where the FeFETs with *x* = 0, 0.1, and 0.2 had wider memory windows than those with *x* = 0.5 and 1. The influence of annealing temperature for C_0.1_S_0.9_BT poly-crystallization was also investigated using the EBSD method.

## 1. Introduction

**Figure 1:**
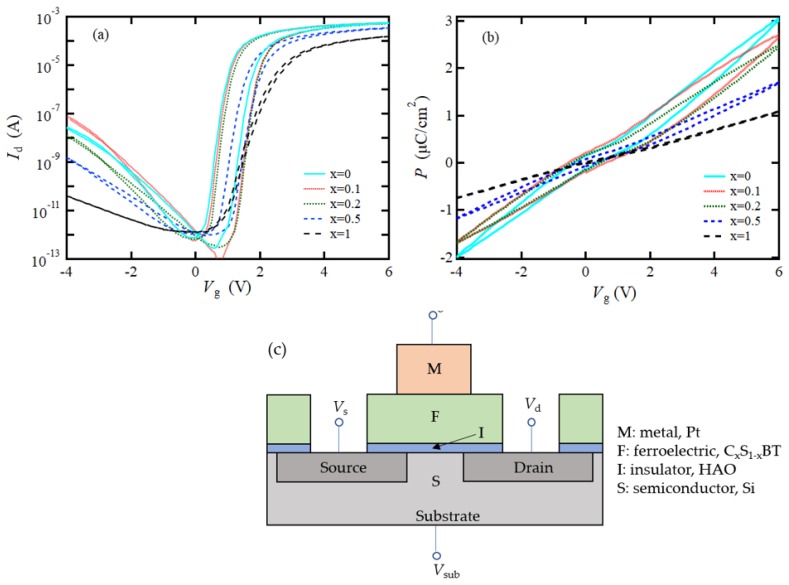
(**a**) Drain current (*I*_d_) vs. gate voltage (*V*_g_)curves, modified from [19]; (**b**) Polarization (*P*) as a function of gate voltage of C_x_S_1–x_BT ferroelectric-gate field-effect transistors (FeFETs) with *x* = 0, 0.1, 0.2, 0.5, and 1. C_x_S_1–x_BT crystallization annealing were performed in O_2_ ambient for 30 min at 813 °C for *x* = 0 and at 800 °C for the others. (**c**) Schematic drawing with voltage terminals for the metal-ferroelectric-insulator-semiconductor (MFIS) type FeFET.

As a memory device, the ferroelectric-gate field-effect transistor (FeFET) has attracted much interest [1,2,3,4,5,6,7]. It has many features, such as non-volatile memory function, voltage driven write operation, nondestructive read operation, and possible compact 4*F*^2^ cell size (*F*: feature size) [1,3]. FeFETs can be applied not only to NAND flash memories intended for large scale storage [8,9,10,11], but also to embedded flash memories with logic devices for portable and other IOT (internet of things) devices with low power dissipation and low voltage operation [12,13,14]. The FeFET in this paper is a type of metal-ferroelectric-insulator-semiconductor (MFIS). The FeFET is shown schematically in Figure 1. MFIS FeFETs with long retention and high endurance were realized for the first time using Pt/SrBi_2_Ta_2_O_9_(SBT)/Hf–Al–O(HAO)/Si gate stacks, where the HAO is (HfO_2_)_y_(Al_2_O_3_)_1–y_, often with *y* = 0.75 [15,16,17]. One-month-long retention was demonstrated by a self-aligned gate FeFET with Pt/SBT/HAO/Si [18]. In 2013, calcium doped SBT, Ca_x_Sr_1–x_Bi_2_Ta_2_O_9_ or C_x_S_1–x_BT, was introduced as a ferroelectric material in MFIS FeFETs that attained larger memory windows of the FeFETs than the conventional SBT [19]. After the FeFETs with C_x_S_1–x_BT were developed, 3.3 V-write-voltage FeFETs [14] and 100-nm metal-gate FeFETs [20] were demonstrated. Write performances of FeFETs were precisely investigated by applying pulse voltages [21]. Memory-cell properties for new ferroelectric NOR flash memories were examined [22].

Ferroelectricity of materials strongly depends on the crystallinity, which has been conventionally characterized by X-ray diffraction (XRD) [23,24]. Recently, the electron backscatter diffraction (EBSD) method was developed into an important technique for metallography [25] and was also used to characterize the ferroelectric materials [26,27]. In this study, the EBSD is applied for the first time in order to discuss the relevance of the nonvolatile-memory-cell performances of the FeFETs with the ferroelectric crystallinity of the C_x_S_1–x_BT hidden inside the MFIS gate stacks. The EBSD method exhibits a two-dimensional map of the C_x_S_1–x_BT grains, whose size and crystal orientation can be understood visually. The minimum size detectable by the EBSD is as small as 10 nm, which can be observed in situ by the field-emission-type scanning electron microscope (FESEM) [25]. The spatial resolution of the EBSD is much higher than that of XRD. With downsizing of ferroelectric devices [20,28], micro area analysis of the ferroelectric layers would be very helpful to understand the quality and achieve better device performance. In this work, the EBSD characterizations of C_x_S_1–x_BT layers in the FeFETs were performed after the manufacturing processes were completed and the electrical properties were identified. In the processes, the whole MFIS stacks were annealed for the C_x_S_1–x_BT crystallization with the Pt electrodes formed on the C_x_S_1–x_BT by photo-lithography and ion-beam etching [19]. Therefore, grain size and orientation of the polycrystalline C_x_S_1–x_BT underneath the gate-electrode Pt could not be directly observed. How to remove the Pt layer was an important technique in this work.

## 2. Experimental

### 2.1. Fabrication of FeFETs and Electrical Characterization

*P*-type Si substrates, with *n*^+^ source and drain regions formed in advance, were used to fabricate *n*-channel MFIS FeFETs with Pt/C_x_S_1–x_BT/(HAO)/Si gate stacks [19]. The thicknesses of HAO, C_x_S_1–x_BT, and Pt were 7 nm, 200 nm, and 200 nm, respectively. HAO and C_x_S_1–x_BT were deposited by pulse laser deposition. Pt was deposited by electron-beam evaporation. Further details of the fabrication process were reported elsewhere [19]. The metal gate length (*L*) was 10 μm, and the gate width (*W*) was 200 μm [19]. Here, two groups of FeFETs were investigated by EBSD. In the first group, *x* was varied. Namely, C_x_S_1–x_BT FeFETs with *x* = 0 (SBT), *x* = 0.1, *x* = 0.2, *x* = 0.5, and *x* = 1 (CaBi_2_Ta_2_O_9_, CBT), were characterized. The SBT FeFET was annealed at 813 °C. The other C_x_S_1–x_BT FeFETs were annealed at 800 °C. In the second group, *x* was fixed at *x* = 0.1 and the C_0.1_S_0.9_BT FeFETs were annealed at nine different temperatures in O_2_ ambient, which were 748, 755, 763, 775, 788, 800, 813, 823, and 833 °C. All the temperatures were set-temperatures, which were about 10 °C higher than the real sample temperatures according to a temperature sensor. The sensor was an *R*-type thermocouple buried in a 20 mm^2^ diced Si wafer. Drain current versus gate voltage (*I*_d_–*V*_g_) curves were measured using an Agilent 4156C transistor analyzer. Memory window (MW) was defined as the difference of the threshold voltages when *I*_d_/*W* = 10^−8^ A/μm on the *I*_d_–*V*_g_ hysteresis loops [19]. Polarization (*P*) versus gate voltage (*P*–*V*_g_) hysteresis curves were obtained using the virtual ground mode of the Radiant RT6000S ferroelectric test system.

### 2.2. EBSD Sample Preparation

High quality Pt/C_x_S_1–x_BT/HAO/Si FeFETs were manufactured successfully with good reproducibility [19]. For crystallizing the C_x_S_1–x_BT, the whole MFIS stacks were annealed. Due to the coverage by the Pt gate metal, the ferroelectric C_x_S_1–x_BT film was hidden in the MFIS stacks. To observe the C_x_S_1–x_BT by EBSD, therefore, the Pt layer was removed using polishing cloths with 0.25 μm-size fine diamond pastes of SCAN-DIA. After the polishing, the samples were ultrasonically cleaned in acetone and in deionized water. Although the polishing made linear scratches to some extent on the surface of the C_x_S_1–x_BT layer, such scratches had no influence on crystal-orientation characterization by EBSD.

### 2.3. EBSD Scanning

An EBSD system (EDAX-TSL, AMETEK, Inc., Berwyn, PA, USA) was installed in the FESEM (JSM-7001F, JEOL Ltd., Tokyo, Japan). During EBSD scanning, the samples were kept 70° tilted from the horizontal position in order to optimize the fraction of electrons scattered and the contrast in the diffraction pattern. When the electron beam hit the sample surface region, some of the scattered electrons agreed with the Bragg’s law and were diffracted from lattice planes. The diffraction patterns in the space, which are well known as Kikuchi bands, were captured by a phosphor screen equipped inside the FESEM. The Kikuchi band of every sample point was collected by the EBSD software and outputted into EBSD maps after Miller indexing. The crystal structure of C_X_S_1–x_BT is orthorhombic (*A*21*am*) [29]. However, since the EBSD software could hardly identify the very small difference of *a* and *b*, we assumed it to be a pseudo-tetragonal structure (I4/mmm), with *a* = *b* = 0.5523 nm and *c* = 2.5026 nm, to index the Kikuchi patterns [27], where *a*, *b*, and *c* were the lattice parameters of the crystal unit cell. During the scanning in this work, the FESEM accelerating voltage, work distance, and magnification were set to 15 kV, 25 mm, and 20,000, respectively. The scanning area and step were 4.4 × 6.6 μm^2^ and 15 nm, respectively. The EBSD is a good method to know the relationship between electrical properties and crystal orientations of ferroelectric materials [27].

## 3. Results and Discussion

### 3.1. Electrical Properties of C_x_S_1–x_BT FeFETs with Varying x

The electrical properties of Pt/C_x_S_1–x_BT/HAO/Si FeFETs with *x* = 0, 0.1, 0.2, 0.5, and 1 were reported in our former work [19]. *I*_d_–*V*_g_ curves are shown in Figure 1a, with *V*_g_ scanning from −4 V to 6 V, and then back to −4 V. During the scanning, the drain voltage (*V*_d_) was set to 0.1 V. The source voltage (*V*_s_) and substrate voltage (*V*_sub_) were kept to zero. Drawing directions of the *I*_d_–*V*_g_ hysteresis loops were counter-clockwise in the cases of x = 0, 0.1, 0.2, and 0.5 V, indicating that the origins of the loops were ferroelectric. MWs of the C_x_S_1–x_BT FeFETs were 0.75 V (*x* = 0, namely, SBT), 0.89 V (*x* = 0.1), 0.84 V (*x* = 0.2), and 0.43 V (*x* = 0.5). In the case of CBT (*x* = 1), the MW was almost zero, but, more exactly, the loop direction was clockwise, indicating that the CBT was not ferroelectric and that the CBT FeFET showed weak charge-injection behavior in the *I*_d_–*V*_g_ measurement.

The polarization, *P*, of the C_x_S_1–x_BT FeFETs was also measured as a function of *V*_g_, as shown in Figure 1b. During the *P*–*V*_g_ measurements, *V*_d_, *V*_s_, and *V*_sub_ were set to 0 V. Note that the *V*_g_ was applied across all the stacked layers, which included C_x_S_1–x_BT, HAO, and the interfacial layer between HAO and Si, and the surface potential of the silicon. While *V*_g_ changed between −4 V and 6 V, half the peak-to-peak amplitudes of polarization (*P*_hppa_) [21,30], were 2.56 μC/cm^2^ (*x* = 0), 2.22 μC/cm^2^ (*x* = 0.1), 2.12 μC/cm^2^ (*x* = 0.2), 1.46 μC/cm^2^ (*x* = 0.5), and 0.93 μC/cm^2^ (*x* = 1). The MWs on *P*–*V*_g_ curves, i.e., the voltage differences when *P* = 0, were 0.72 V (*x* = 0), 1.18 V (*x* = 0.1), 0.86 V (*x* = 0.2), 0.56 V (*x* = 0.5), and 0.1 V (*x* = 1). Drawing directions of the *P*–*V*_g_ hysteresis loops for all cases were counter-clockwise. As a non-volatile memory FeFET cell, a large coercive field of the C_x_S_1–x_BT is preferred rather than a spontaneous or remnant polarization if an appropriate *P*_hppa_ (typically 2.5 μC/cm^2^) is attained [30]. 

### 3.2. EBSD Characterization Results for C_x_S_1–x_BT FeFETs with Varying x

**Figure 2:**
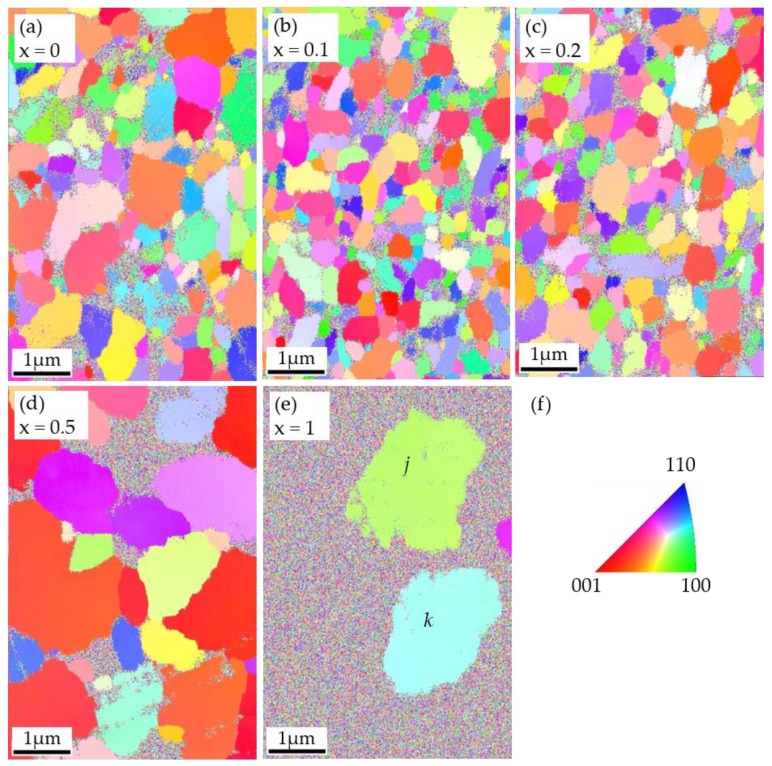
Electron backscatter diffraction (EBSD) inverse pole figure map of C_x_S_1–x_BT layer in the FeFET stacks with (**a**) *x* = 0; (**b**) *x* = 0.1; (**c**) *x* = 0.2; (**d**) *x* = 0.5; and (**e**) *x* = 1; (**f**) Color coded unit triangle.

**Figure 3:**
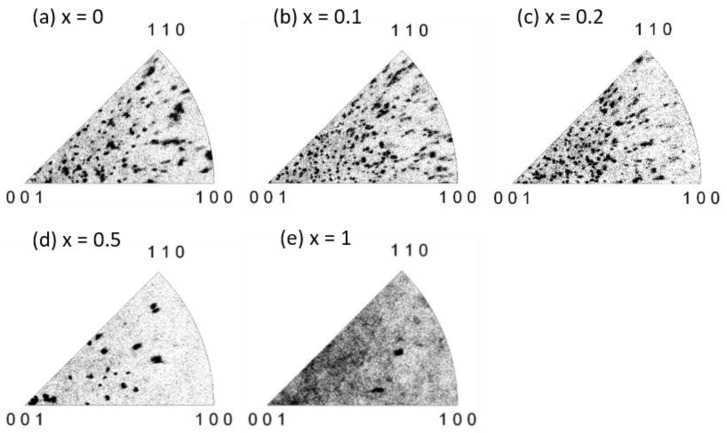
EBSD inverse pole figure unit triangle of C_x_S_1–x_BT layer in the FeFET stacks, with (**a**) *x* = 0; (**b**) *x* = 0.1; (**c**) *x* = 0.2; (**d**) *x* = 0.5; and (**e**) *x* = 1.

The EBSD scanning results are shown in Figure 2 and Figure 3. Figure 2a–e are inverse pole figure maps, where every point of the C_x_S_1–x_BT was shaded with a color guided by the color code unit triangle according to its orientation (Figure 2f). In the map (Figure 2), the different colors represent the different orientations. The red color point designated as (001) means that the *c*-axis of the grain is parallel to the film normal. The green color point designated as (100) means that the *a*-axis of the grain is parallel to the film normal. When the points with the same crystal orientation are accumulated together and make an area of one color, this is a grain of C_x_S_1–x_BT observed by EBSD. A neutron diffraction showed that SBT and CBT have spontaneous polarization along the *a*-axis [31]. Electrical measurements of polarization versus electric field (*P*–*E*) curves of epitaxial SBT thin films supported the *a*-axis directed spontaneous polarization [32,33]. Hence the direction of spontaneous polarization of C_x_S_1–x_BT can be regarded as the *a*-axis, and the more green color in the EBSD map (Figure 2f) can be attributed to larger polarization. Figure 3 indicates the unit triangles of inverse pole figures, in which one black point represents one unique orientation of the C_x_S_1–x_BT.

**Figure 4:**
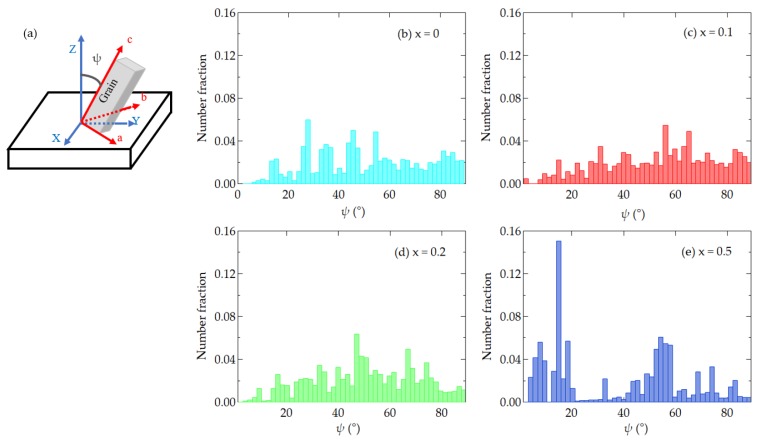
(**a**) Definition of angle *ψ*, and EDSD analyses of crystal orientation distribution represented as a function of *ψ* of the C_x_S_1–x_BT layers in the FeFETs with (**b**) *x* = 0; (**c**) *x* = 0.1; (**d**) *x* = 0.2; and (**e**) *x* = 0.5. In each Figure of (**b**–**e**), integration of number fraction as a function of *ψ* makes 1.0.

**Figure 5:**
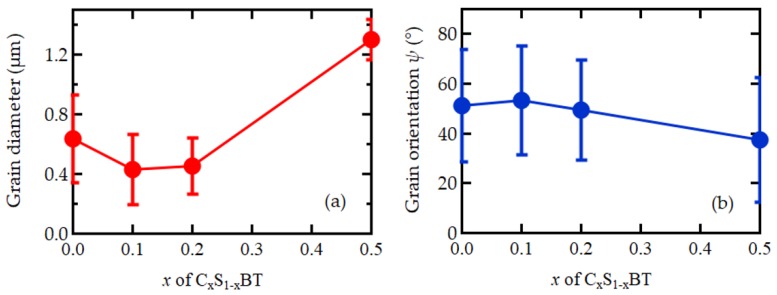
Average values of (**a**) grain sizes and (**b**) crystal orientations of C_x_S_1–x_BT with *x* = 0, 0.1, 0.2, and 0.5. Error bar lengths are twice the standard deviations.

The crystal orientation data of grains can also be characterized by the angle *ψ* (Figure 4a) between the film normal and the grain *c*-axis. Here the EBSD distribution density was integrated over with respect to the other Euler angles. Orientation distributions as a function of *ψ* for C_x_S_1–x_BT FeFETs with *x* = 0, 0.1, 0.2, and 0.5 are shown in Figure 4b–e, respectively. The crystal orientations in the SBT (*x* = 0), C_0.1_S_0.9_BT (*x* = 0.1), and C_0.2_S_0.2_BT (*x* = 0.2) are broadly distributed in the range of 0° < *ψ* ≤ 90°. On the other hand, the distribution of C_0.5_S_0.5_BT (*x* = 0.5) shows a large distribution below 20°, representing the near-*c*-axis oriented crystallization. Average values of the grain diameters (Figure 2a–d) and crystal orientation angles (Figure 4b–e) of the C_x_S_1–x_BT with *x* = 0, 0.1, 0.2, and 0.5 were statistically calculated with the standard deviations as shown in Figure 5a,b. 

### 3.3. Energy Dispersive X-ray Spectroscopy Characterization of CBT

**Figure 6:**
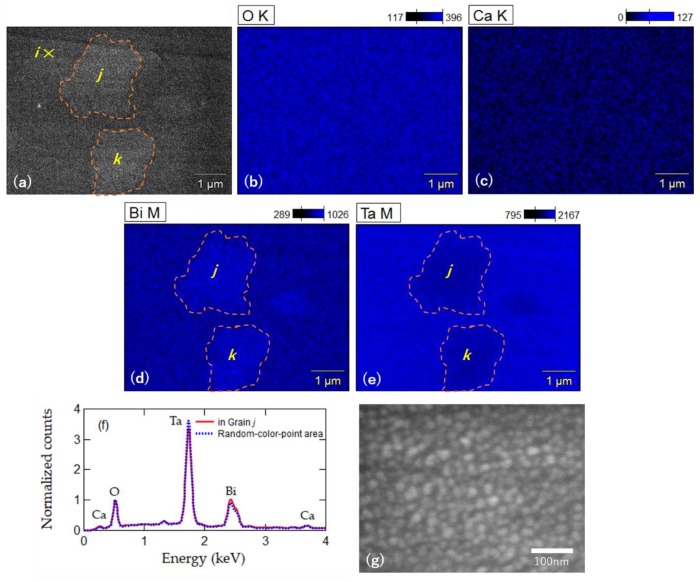
(**a**) Gray-colored energy dispersive X-ray spectroscopy (EDX) map of the integrated counts. The grains *j* and *k* are the same grains appearing in Figure 2e. The EDX element mapping of (**b**) O; (**c**) Ca; (**d**) Bi; and (**e**) Ta. Each color bar at the top right in (**b**–**e**) represents a scale of intensity counts of the element. The color for the zero intensity would be black; (**f**) EDX spectra both on the area *j* and on the multi-color-dot area, where the counts are normalized to the O peak; (**g**) SEM image at the spot *i*, shown in (**a**).

In Figure 2, there were noisy areas composed of dots with multiple colors. Such noisy areas existed only near the grain boundaries in the C_x_S_1–x_BT maps of *x* = 0, 0.1, and 0.2, but appeared more in the maps of *x* = 0.5. In the case of CBT, more than half the area was noisy. As an experimental fact, no Kikuchi bands appeared on these noisy multi-color-dot areas during the EBSD scanning. In order to consider the origin of the noises, we made an energy dispersive X-ray spectroscopy (EDX) characterization for the CBT as shown in Figure 2e. EDX (Noran System 7, Thermo Scientific) was equipped with the same FESEM as the EBSD was. The observed area was the same as the area used for the EBSD characterization. Figure 6a–e show the EDX mapping results. Figure 6a is the gray-colored intensity map of the integrated counts. Figure 6b–e are the separated maps of the elements, O, Ca, Bi, and Ta. The surrounded areas *j* and *k* shown in Figure 6a,d,e are positioned at the same grains *j* and *k* in Figure 2e by the EBSD. Maps for Bi and Ta had shades of colors with the same shapes of the grains *j* and *k* (Figure 6d,e), whereas those for O and Ca had no features with uniform colors (Figure 6b,c). According to the EDX maps for the Bi and Ta (Figure 6d,e), the noisy multi-color-dot area outside *j* and *k* included less Bi and more Ta than the area inside *j* and *k*. The EDX spectra of the local two spots, inside *j* and outside *j* and *k*, were investigated using ten-times as large a magnification as that used in Figure 6a–e. As shown in Figure 6f, the spot outside *j* and *k* exhibited lower Bi and higher Ta intensity peaks than the spot inside *j*, while both spots had exactly overlapped Ca peaks. A magnified SEM image at the position *i* in Figure 6a indicated that the spot outside *j* and *k*, which was in the multi-color-dot area in Figure 2e, consisted of tiny grains as small as 20 nm (Figure 6g). By these experimental results, there are possible two explanations for the multi-color-dot areas. The first explanation is that grains in the areas were crystalized with random orientations, but they were as small as the resolution limit of EBSD. Hence Kikuchi bands could not be observed. The second one is that grains were not well crystalized yet. From the latter view point, a possible phase was Bi-substituted CaTa_2_O_6_ on the multi-color-dot area, whose crystallization temperature was much higher than the present annealing temperature of about 800 °C [34,35]. Further investigation in the future will provide a sure solution. 

### 3.4. X-ray Diffraction for C_x_S_1–x_BT

**Figure 7:**
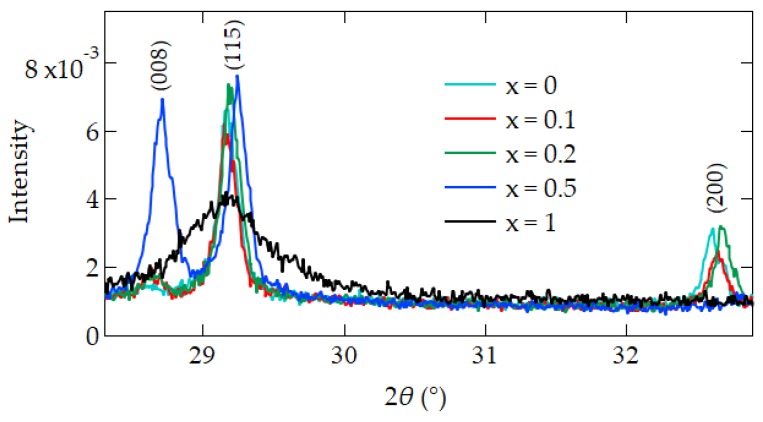
X-ray diffraction (XRD) profiles of C_x_S_1–x_BT with (**a**) *x* = 0; (**b**) *x* = 0.1; (**c**) *x* = 0.2 (**d**) *x* = 0.5; and (**e**) *x* = 1. The intensities were normalized to the Si (400) peak intensity. Modified from [19].

The XRD spectra of C_x_S_1–x_BT are shown in the Figure 7. Samples of Pt/CSBT/HAO/Si with non-patterned Si were used [19]. The SBT and C_x_S_1–x_BT with *x* = 0.1 and 0.2 have narrow and strong non-*c*-axis peaks of (115) and (200). They do not have the *c*-axis oriented (008) peak. On the other hand, C_0.5_S_0.5_BT shows the (008) *c*-axis peak as well as the (115) peak but does not have the (200) peak. The XRD measurements of the *c*-axis orientation more in the C_0.5_S_0.5_BT than in the C_x_S_1–x_BT with *x* = 0, 0.1, and 0.2 are in good agreement with the EBSD characterization, which showed the C_0.5_S_0.5_BT orientated near the *c*-axis direction (Figure 4e). The XRD of the CBT seemed insufficiently crystalized because the (115) peak got weaker and broader than those of SBT and C_x_S_1–x_BT of x = 0.1 and 0.2, and the (008) and (200) peaks did not appear. The insufficient crystallization of the CBT was supported by EBSD observation of a major area occupied by multi-color dots (Figure 2e).

### 3.5. Comparison of Electrical Properties with Results of EBSD and Other Characterizations

In the EBSD maps of Figure 2a–c, many grains with rather uniform size and non-c-axis orientation were recognized on SBT, C_0.1_S_0.9_BT, and C_0.2_S_0.8_BT layers. As a function of the angle *ψ* between the film normal and the grain c-axis (Figure 4a), the crystal orientations of the SBT, C_0.1_S_0.9_BT, and C_0.2_S_0.8_BT layer were broadly distributed in the wide range of 0° < *ψ* ≤ 90° as shown in Figure 4b–d. In fact, the average values of the *ψ* were about 50° regarding the C_x_S_1–x_BT with *x* = 0, 0.1 and 0.2 (Figure 5b). The XRD profiles of the C_x_S_1–x_BT with *x* = 0, 0.1, and 0.2 in Figure 7, which have the (200) and (115) peaks without the (008) peak, are consistent with the wide range *ψ* distributions of the crystal orientations observed by the EBSD. Since spontaneous polarization of C_x_S_1–x_BT is expected to exist along the *a*-axis, inclusion of the film-normal *a*-axis component is required to obtain polarization versus electric field (*P*–*E*) hysteresis curves of the C_x_S_1–x_BT, where the *E* is applied across the C_x_S_1–x_BT film. Regarding the C_x_S_1–x_BT with *x* = 0, 0.1, and 0.2, the wide range *ψ* distributions observed by EBSD (Figure 4b–d) agreed with the large MWs of the C_x_S_1–x_BT FeFETs (Figure 1a) due to the inclusion of the *a*-axis components in the C_x_S_1–x_BT film-normal directions, which were also supported by Figure 7. 

In the case of the C_0.5_S_0.5_BT FeFET, the EBSD map (Figure 2d) showed many grains painted in nearly red colors, which indicated *c*-axis-preferred crystal orientations. Figure 4e also indicated the *c*-axis-preferred crystal orientations of the C_0.5_S_0.5_BT by the large number of fractions below 20°. As shown in Figure 5b, the average value of *ψ* was 37°, which was about 13° lower than those of C_x_S_1–x_BT with *x* = 0, 0.1, and 0.2. In the XRD profile of the C_0.5_S_0.5_BT in Figure 7, the inclusion of *c*-axis crystal orientation was confirmed by the (008) peak. Figure 7 indicated that the C_0.5_S_0.5_BT had the (115) peak but had no (200) peak corresponding to the *a*-axis orientation. The *c*-axis-preferred crystal orientations are expected to show very small ferroelectric-polarization switching by imposing bipolar *V*_g_ pulses. Actually, the C_0.5_S_0.5_BT FeFET had smaller MW than the C_x_S_1–x_BT with *x* = 0, 0.1, and 0.2 (Figure 1a). The increase of a noisy multi-color-dot area of the C_0.5_S_0.5_BT (Figure 2d) can be another reason for the small MW of the C_0.5_S_0.5_BT FeFET because it would be non-ferroelectric in the area. In the C_0.5_S_0.5_BT FeFET, therefore, the ferroelectric behavior was weakened, resulting in the memory window 0.43 V being much narrower than those for the FeFET of x = 0, 0.1, and 0.2 (Figure 1a). 

In the CBT FeFET case, more than half of the EBSD map was occupied by the multi-color-dot areas (Figure 2e). This suggests insufficient crystallization of such areas, in which no ferroelectricity is expected. The XRD profile in Figure 7 showed only a weak and broad peak at the (115) position, which also indicated the insufficient crystallization of the CBT. The non-ferroelectric *I*_d_–*V*_g_ and *P*–*V*_g_ curves of the CBT FeFET in Figure 1a can be understood by the EBSD and XRD investigations. 

### 3.6. EBSD Characterization for C_0.1_S_0.9_BT with Various Annealing Temperatures

**Figure 8:**
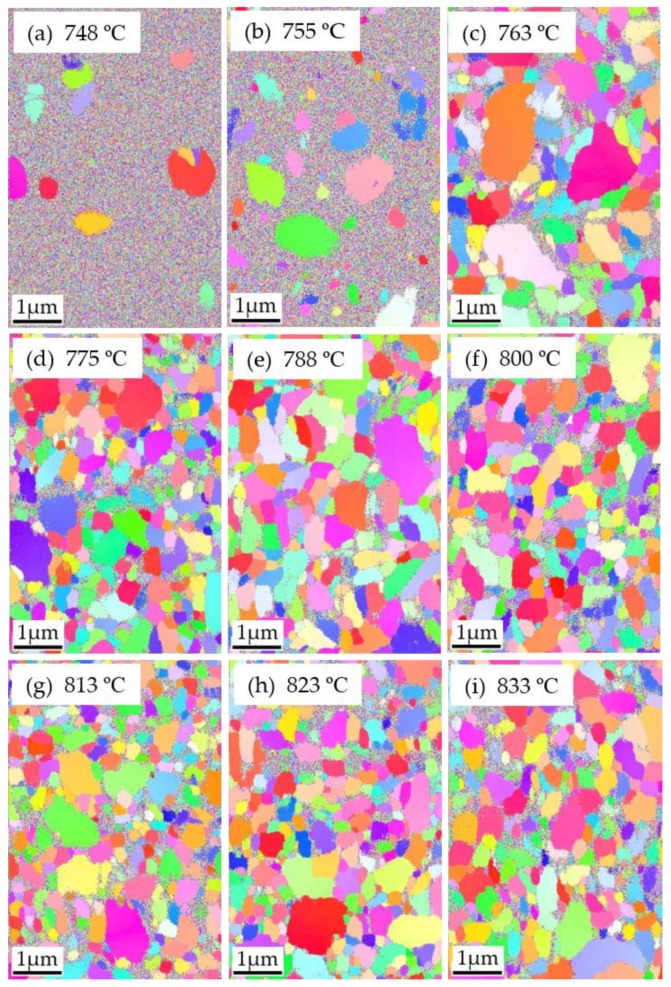
EBSD map of C_0.1_S_0.9_BT FeFETs with different annealing temperature, (**a**) 748 °C, (**b**) 755 °C, (**c**) 763 °C, (**d**) 775 °C, (**e**) 788 °C, (**f**) 800 °C, (**g**) 813 °C, (**h**) 823 °C, and (**i**) 833 °C. Color coded unit triangle is the same as Figure 2f.

The EBSD characterization was also performed to investigate the influence of annealing temperature (*T*_a_) on the C_0.1_S_0.9_BT crystallization. Figure 8 shows the EBSD map of C_0.1_S_0.9_BT in the FeFET annealed at *T*_a_ = 748, 755, 763, 775, 788, 800, 813, 823, and 833 °C. For *T*_a_ = 748 °C, several grains were found, but noisy multi-color-dot areas covered most parts of the scanning area. For *T*_a_ = 755 °C, although the multi-color-dot areas still occupied more than half the map, grain size and number were increased in comparison with those for *T*_a_ = 748 °C. In the case of *T*_a_ ≥ 763 °C, grains with certain crystal orientations covered the most scanning area. In the temperature range from *T*_a_ = 775 °C to 833 °C (Figure 8c–i), the grains had uniform sizes and random crystal orientations. Multi-color-dot areas were located only on the grain boundaries in Figure 8c–i, without significant differences among the figures. 

**Figure 9:**
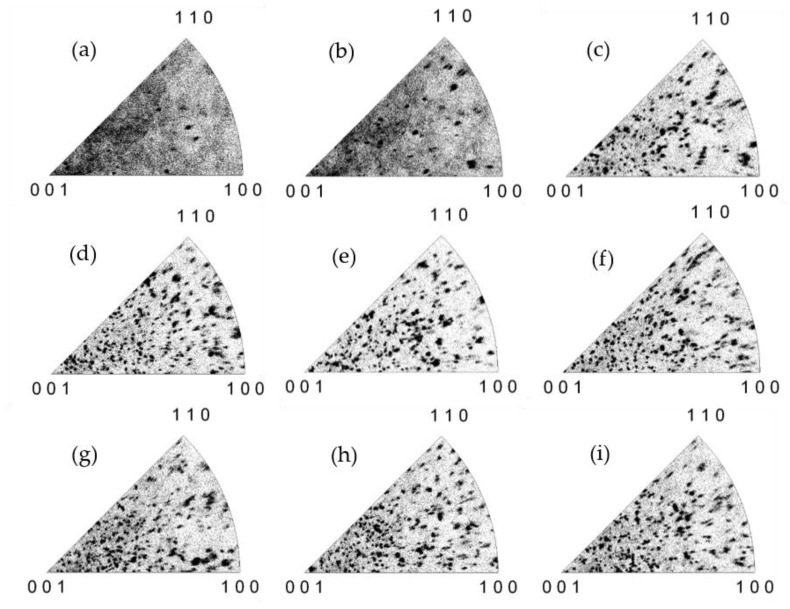
EBSD inverse pole figure of C_x_S_1–x_BT FeFETs with different annealing temperature, (**a**) 748 °C, (**b**) 755 °C, (**c**) 763 °C, (**d**) 775 °C, (**e**) 788 °C, (**f**) 800 °C, (**g**) 813 °C, (**h**) 823 °C, and (**i**) 833 °C.

**Figure 10:**
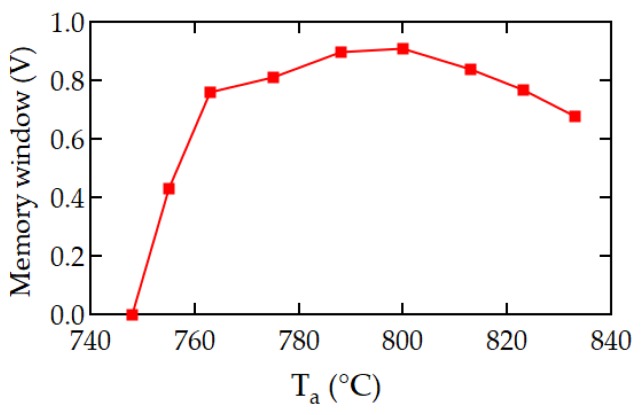
Memory windows vs. annealing temperature curve of C_x_S_1–x_BT (*x* = 0.1) FeFETs. Modified from [19].

The inverse polar figures (Figure 9a–i) showed a similar tendency without significant differences from Figure 8a–i. The memory windows of these FeFETs that were reported [19] are shown in the Figure 10 as a function of *T*_a_. The MW of the C_0.1_S_0.9_BT FeFET with *T*_a_ = 748 °C was 0 V. The MW with *T*_a_ = 755 °C was 0.43 V. With further increases of *T*_a_ from 763 °C to 813 °C, the MW became as large as 0.8 V–0.9 V. More exactly, at *T*_a_ = 788 °C and 800 °C the MWs exhibited the maximum. After exceeding the maximum region, the MWs were gradually decreased. MW = 0.68 V was obtained at *T*_a_ = 833 °C. 

The increasing MW with increasing *T*_a_ from 748 °C to 763 °C can be explained by the increase of the size and frequency of grains and by the decrease of the noisy multi-color-dot areas. Crystallinity may be improved with further increases of *T*_a_. The FeFETs showed the maximum MW in the *T*_a_ range from 788 °C to 800 °C. The decreases of MWs at *T*_a_ > 800 °C may be caused by the increase of a SiO_2_-like interfacial layer (IL) grown between the I layer and the S surface in the MFIS of the FeFETs. As shown in [14,16,17,19], during the annealing for the C_x_S_1–x_BT crystallization, the IL is grown to normally a few nano-meters-thick. The IL may get thick as the *T*_a_ increases from 800 °C to 833 °C. In the MFIS, three capacitances of F, I, and IL and a capacitance of S (appearing when the depletion layer is formed) are connected in series and share the *V*_g_. The voltage across the C_x_S_1–x_BT is reduced when the IL becomes thick because the voltage across the IL is increased. With the voltage decrease across the C_x_S_1–x_BT, the MW may become narrow as *T*_a_ is increased from 800 °C to 833 °C. 

## 4. Conclusions

The EBSD method was used to characterize the Ca_x_Sr_1–x_Bi_2_T_2_O_9_ layer inside the FeFET stacks. The distributions of grain size and orientation of C_x_S_1–x_BT with *x* = 0 (SBT), 0.1, 0.2, 0.5, and 1 (CBT) were presented in the inverse polar figure maps and the unit triangles of EBSD. The grain size and crystal orientation distributions depended on the content *x* of C_x_S_1–x_BT. There were more uniform and smaller grains with random orientation in SBT, C_0.1_S_0.9_BT, and C_0.2_S_0.8_BT. In C_0.5_S_0.5_BT, grains got larger and the major grains were orientated near the *c*-axis direction. Since spontaneous polarization exists along the *a*-axis, these EBSD results explain well the reason that the memory windows of SBT, C_0.1_S_0.9_BT, and C_0.2_S_0.8_BT FeFETs were much wider than that of the C_0.5_S_0.5_BT FeFET. EBSD investigation also revealed the existence of noisy multi-color-dot areas on which no Kikuchi-bands appeared, indicating that the areas were non-ferroelectric. EBSD characterizations of the C_0.1_S_0.9_BT FeFETs with various annealing temperatures from *T*_a_ = 748 to 833 °C were performed. As the *T*_a_ was raised from 748 °C toward 763 °C, grain size and number increased, and the noisy multi-color-dot areas decreased. When *T*_a_ was increased from 763 °C to 833 °C, the EBSD maps showed the C_0.1_S_0.9_BT mostly composed of grains with rather uniform sizes and random crystal orientations. Good consistency among the EBSD investigations of the C_x_S_1–x_BT and memory window widths of the C_x_S_1–x_BT FeFETs could be discussed.

## References

[1] Tarui Y., Hirai T., Teramoto K., Koike H., Nagashima K. (1997). Application of the ferroelectric materials to ULSI memories. Appl. Surf. Sci..

[2] Scott J.F. (2000). Ferroelectric Memories. Advanced Microelectronics.

[3] Sakai S., Takahashi M. (2010). Recent progress of ferroelectric-gate field-effect transistors and applications to nonvolatile logic and FeNAND flash memory. Materials.

[4] Böscke T.S., Müller J., Bräuhaus D., Schröder U., Böttger U. Ferroelectricity in hafnium oxide: CMOS compatible ferroelectric field effect transistors. Proceedings of the 2011 IEEE International Electron Devices Meeting.

[5] Sakai S., Zhang X.Z., Hai L.V., Zhang W., Takahashi M. Downsizing and memory array integration of Pt/SrBi_2_Ta_2_O_9_/Hf-Al-O/Si ferroelectric-gate field-effect transistors. Proceedings of the 2012 12th Annual Non-Volatile Memory Technology Symposium.

[6] Müller J., Polakowski P., Mueller S., Mikolajick T. (2014). Ferroelectric hafnium oxide based materials and devices: Assessment of current status and future prospects. ECS Trans..

[7] Okuyama M. (2016). Features, principles and development of ferroelectric-gate field-effect transistors. Ferroelectric-Gate Field Effect Transistor Memories: Device Physics and Applications.

[8] Sakai S., Takahashi M., Takeuchi K., Li Q.-H., Horiuchi T., Wang S., Yun K.-Y., Takamiya M., Sakurai T. Highly scalable Fe(ferroelectric)-NAND cell with MFIS(metal-ferroelectric-insulator-semiconductor) structure for sub-10nm tera-bit capacity NAND flash memories. Proceedings of the 2008 Joint Non-Volatile Semiconductor Memory Workshop and International Conference on Memory Technology and Design.

[9] Wang S., Takahashi M., Li Q.-H., Takeuchi K., Sakai S. (2009). Operational method of a ferroelectric (Fe)-NAND flash memory array. Semicond. Sci. Technol..

[10] Zhang X., Takahashi M., Sakai S. (2012). FeFET logic circuits for operating a 64 kb FeNAND flash memory array. Integr. Ferroelectr..

[11] Zhang X., Takahashi M., Takeuchi K., Sakai S. (2012). 64 kbit ferroelectric-gate-transistor-integrated NAND flash memory with 7.5 V program and long data retention. Jpn. J. Appl. Phys..

[12] Takahashi M., Horiuchi T., Li Q.-H., Wang S., Yun K.-Y., Sakai S. (2008). Basic operation of novel ferroelectric CMOS circuits. Electron. Lett..

[13] Takahashi M., Wang S., Horiuchi T., Sakai S. (2009). FeCMOS logic inverter circuits with nonvolatile-memory function. IEICE Electron. Express.

[14] Zhang W., Takahashi M., Sasaki Y., Kusuhara M., Sakai S. (2017). 3.3 V write-voltage Ir/Ca_0.2_Sr_0.8_Bi_2_Ta_2_O_9_/HfO_2_/Si ferroelectric-gate field-effect transistors with 10^9^ endurance and good retention. Jpn. J. Appl. Phys..

[15] Sakai S., Ilangovan R. (2004). Metal-ferroelectric-insulator-semiconductor memory FET with long retention and high endurance. IEEE Electron Device Lett..

[16] Sakai S., Ilangovan R., Takahashi M. (2004). Pt/SrBi_2_Ta_2_O_9_/Hf-Al-O/Si field-effect-transistor with long retention using unsaturated ferroelectric polarization switching. Jpn. J. Appl. Phys..

[17] Sakai S., Takahashi M., Ilangovan R. Long-retention ferroelectric-gate FET with a (HfO_2_)_x_(Al_2_O_3_)_1−x_ buffer-insulating layer for 1T FeRAM. Proceedings of the 2004 IEDM Technical Digest. IEEE International Electron Devices Meeting.

[18] Takahashi M., Sakai S. (2005). Self-aligned-gate metal/ferroelectric/insulator/semiconductor field-effect transistors with long memory retention. Jpn. J. Appl. Phys..

[19] Zhang W., Takahashi M., Sakai S. (2013). Electrical properties of Ca_x_Sr_1−x_Bi_2_Ta_2_O_9_ ferroelectric-gate field-effect transistors. Semicond. Sci. Technol..

[20] Hai L.V., Takahashi M., Zhang W., Sakai S. (2015). 100-nm-size ferroelectric-gate field-effect transistor with 10^8^-cycle endurance. Jpn. J. Appl. Phys..

[21] Sakai S., Zhang W., Takahashi M. Dynamic analog characteristics of 10^9^ cycle-endurance low-voltage nonvolatile ferroelectric-gate memory transistors. Proceedings of the 2017 IEEE 9th International Memory Workshop.

[22] Takahashi M., Zhang W., Sakai S. High-endurance ferroelectric NOR flash memory using (Ca,Sr)Bi_2_Ta_2_O_9_ FeFETs. Proceedings of the 2018 IEEE 10th International Memory Workshop.

[23] Watanabe H., Mihara T., Yoshimori H., de Araujo C.A.P. (1995). Preparation of ferroelectric thin films of bismuth layer structured compounds. Jpn. J. Appl. Phys..

[24] Amanuma K., Hase T., Miyasaka Y. (1995). Preparation and ferroelectric properties of SrBi_2_Ta_2_O_9_ thin films. Appl. Phys. Lett..

[25] Humphreys F.J. (2001). Review grain and subgrain characterization by electron backscatter diffraction. J. Mater. Sci..

[26] Koblischka-Veneva A., Mücklich F. (2002). Orientation imaging microscopy applied to BaTiO_3_ ceramics. Cryst. Eng..

[27] Kaibara K., Tanaka K., Uchiyama K., Kato Y., Shimada Y. (2008). Electron backscatter diffraction analysis for polarization of SrBi_2_(Ta,Nb)_2_O_9_ ferroelectric capacitors in submicron small area. Jpn. J. Appl. Phys..

[28] Hai L.V., Takahashi M., Sakai S. Downsizing of ferroelectric-gate field-effect-transistors for ferroelectric-NAND flash memory cells. Proceedings of the 2011 3rd IEEE International Memory Workshop.

[29] Shimakawa Y., Kubo Y., Nakagawa Y., Kamiyama T., Asano H., Izumi F. (1999). Crystal structures and ferroelectric properties of SrBi_2_Ta_2_O_9_ and Sr_0.8_Bi_2.2_Ta_2_O_9_. Appl. Phys. Lett..

[30] Sakai S., Zhang W., Takahashi M. (2017). Method for disclosing invisible physical properties in metal-ferroelectric-insulator-semiconductor gate stacks. J. Phys. D Appl. Phys..

[31] Shimakawa Y., Kubo Y., Nakagawa Y., Goto S., Kamiyama T., Asano H., Izumi F. (2000). Crystal structure and ferroelectric properties of *A*Bi_2_Ta_2_O_9_ (*A* = Ca, Sr, and Ba). Phys. Rev. B.

[32] Ishikawa K., Funakubo H., Saito K., Suzuki T., Nishi Y., Fujimoto M. (2000). Crystal structure and electrical properties of epitaxial SrBi_2_Ta_2_O_9_ films. J. Appl. Phys..

[33] Watanabe T., Sakai T., Funakubo H., Saito K., Osada M., Yoshimoto M., Sasaki A., Liu J., Kakihana M. (2002). Ferroelectric property of *a*-/*b*-axis-oriented epitaxial Sr_0.8_Bi_2.2_Ta_2_O_9_ thin films grown by metalorganic chemical vapor deposition. Jpn. J. Appl. Phys..

[34] Lee H.-J., Kim I.-T., Hong K.S. (1997). Dielectric properties of AB_2_O_6_ compounds at microwave frequencies (A = Ca, Mg, Mn, Co, Ni, Zn, and B = Nb, Ta). Jpn. J. Appl. Phys..

[35] Ferrari C.R., de Camargo A.S.S., Nunes L.A.O., Hernandes A.C. (2004). Laser heated pedestal growth and optical characterization of CaTa_2_O_6_ single crystal fiber. J. Cryst. Growth.

